# Response Rate of Cisplatin Plus Docetaxel as Primary Treatment in Locally Advanced Head and Neck Carcinoma (Squamous Cell Types)

**DOI:** 10.31557/APJCP.2020.21.3.825

**Published:** 2020-03

**Authors:** Maryum Nouman, Ghulam Haider, Neelma Bukhari, Aveen Yousuf, Rabeea Nouman, Mehwish Roshan Shaikh, Shahid Hussain, Bhunisha Pavan, Raja Rahool, Paras Memon, Saima Zahoor, Khalil Mehar, Abdus Sami

**Affiliations:** *Department of Medical Oncology, Jinnah Postgraduate Medical Center, Karachi, Pakistan. *

**Keywords:** Induction chemotherapy, Docetaxel, cisplatin, locally advanced head and neck carcinoma, response rate

## Abstract

**Objective:**

To evaluate the response rate of Cisplatin plus Docetaxel in the treatment of locally advanced head and neck squamous cell carcinomas (HNSCC) at a tertiary care hospital in Karachi, Pakistan**. **

**Materials and Methods:**

It was a longitudinal study, conducted at the Department of Medical Oncology, Jinnah Postgraduate Medical Center, Karachi, Pakistan from December 2018 to June 2019. One hundred patients of age 14-66 years of age of either gender with histologically proven Squamous Cell Carcinoma of Head and Neck, Stage III and IV (locally advanced) with no distant metastases were included in the study. Patients who were declared unresectable by the otolaryngologist and those with delayed appointment for radiation were given 3 cycles of Induction Chemotherapy with Cisplatin and Docetaxel, both at a dose of 75mg/m^2^ 3 weekly. After 3 cycles, CT scan was repeated to assess the clinical response. Those patients who had partial or complete response as per RECIST criteria were re-assessed by the otolaryngologist and were planned for surgery if disease became resectable while other patients were referred for Concurrent Chemo-Radiation Therapy (CCRT). SPSS version 23 was used to analyze data.

**Results:**

The partial response was achieved in majority of the patients after Induction Chemotherapy with Docetaxel and Cisplatin (62%) with a complete response in 12 %. However, 22% showed progression of the disease, and 4% showed stable disease. The most frequent side effects observed were diarrhea (62%) and neutropenia (57%).

**Conclusion:**

Induction chemotherapy with Cisplatin and Docetaxel is a promising regimen with good response and favorable toxicity profile and can be considered as a potentially effective outpatient regimen for locally advanced squamous cell carcinoma of head and neck.

## Introduction

Around the globe head and neck carcinomas (HNCs) is the 6^th^ most common cancer with the incidence of 650,000 cases and 330,000 cases of mortality yearly (Bray et al., 2018). HNCs are mainly seen in men, but the man-to-woman ratio differs globally and by anatomical site with a ratio fluctuating from 2:1 to 4:1 (Lambert et al., 2011; Bray et al., 2013). Most of the HNCs appear in the epithelium of oral cavity, larynx, oropharynx and hypopharynx (Jemal et al., 2007; Boyle and Levin, 2008). 

The overall 5 year survival rate of HNCs patients is estimated as 40-50% (Kozakiewicz and GrzybowskaSzatkowska, 2018). Early stages (stage I and II) of HNCs showed favorable prognosis with increased cure rates of more than 70-90% (Hashibe et al., 2009; Fung and Grandis, 2010) with surgery and radiotherapy alone (WHO, 2014). The patients with locally advanced stage of HNCs (stage III and IV) have been treated with multimodality therapy. In these advanced stages, surgery along with chemoradiotherapy or radiotherapy have been given to the patients for the treatment which depends upon the grade, histology or metastases of tumor in the regional lymph nodes (Adelstein et al., 2017; Brierley et al., 2017). In the meta-analysis, concurrent chemoradiotherapy (CCRT) has showed significant outcome of chemotherapy plus radiation, which gives an absolute of 8% survival benefit at 5 years in HNCs patients (Pignon et al., 2000). Thus CCRT has been considered as standard therapy for locally advanced HNCs but it has complications such as dermatitis, dysphagia and mucositis. CCRT has also increased the odds of bleeding or infection and frequently accompanied by thrombocytopenia and leukopenia (Andresen et al., 2006; Fung and Grandis, 2010; Kozakiewicz and Grzybowska-Szatkowska, 2018).

The role of Induction Chemotherapy (ICT) remains investigational despite tremendous research. For patients with locally advanced squamous cell carcinoma of the head and neck (HNSCC), rapid advancements regarding the choice of induction chemotherapy regimen have been made. There is substantial evidence related to the advantage and superiority of three-drug combination of Docetaxel, Cisplatin, and Fluorouracil (TPF) over the doublet regime of Cisplatin and Fluorouracil (PF) used previously as ICT. docetaxel has shown promising results in recurrent and incurable cases of HNCs (Catimel et al., 1994; Dreyfuss et al., 1996). docetaxel has a longer intracellular half-life, greater affinity for tubulin and promotes microtube stabilization at lower drug concentrations. Docetaxel has distinctive chemical mechanism of action which helps in inducing the mitotic block in proliferating cells (Lavelle et al., 1995; Posner and Lefebvre, 2003). In the clinical trials of phase I and II docetaxel plus cisplatin or fluorouracil (5-FU) for the treatment of locally advanced squamous-cell HNCs, showed prolonged survival and high clinical and pathological response rates (Colevas et al., 2000; Tubiana-Mathieu et al., 2000). In the clinical trials by Colevas et al. and Tubiana et al. of docetaxel with 5-FU showed over all response rates of 24-27% (Colevas et al., 2000; Tubiana-Mathieu et al., 2000). Whereas in the clinical trials of docetaxel with cisplatin, the overall response rates were reported as 33-76% and appeared to be more efficacious combination (Kienzer et al., 1998; Schoffski et al., 1999; Specht et al., 2000; Glisson et al., 2002).

The aim of our study was to evaluate the response rate of cisplatin plus docetaxel as Induction chemotherapy in the treatment of locally advanced HNCs (squamous cell types). This study will help in improving the functional outcomes and cure rates and integration of cisplatin with docetaxel as the potential agents of chemotherapy however this approach might be helpful in both resectable and unresectable HNCs patients. 

## Materials and Methods

It was a longitudinal study, conducted at the department of Medical oncology of Jinnah Postgraduate medical Center from Sept 2018 to Apr 2019. The sample size was estimated using Open Epi online sample size calculator by taking statistics for complete response as 13% in patients of locally advanced squamous cell carcinoma of head and neck treated with docetaxel (Dreyfuss et al., 1996), margin of error as 6.6% and 95% confidence interval, the calculated sample size came out as 100. Non-probability consecutive sampling technique was employed for sample selection. The patients of age 14-66 years of either gender with histological proven squamous cell carcinoma of head and neck of locally advanced stage III and IV with no distant metastases were included in the study. Patients with previous history of chemotherapy and radiotherapy, Eastern Cooperative Oncology Group (ECOG) performance status of 3, known hypersensitivity to drugs, ischemic heart disease, cirrhosis and bleeding tendency were excluded from the study.

The ethical review committee approval was sought before the conduct of study. Informed written and verbal consent was taken from all the patients. Information regarding socio-demographic and clinical factors were obtained from all the patients. Clinically the initial stage of tumor was assessed on CT scan. Patients with locally advanced disease rendered inoperable by otolaryngologist and those whose radiation appointment was very late were given 3 cycles of induction chemotherapy with docetaxel and cisplatin both at a dose of 75 mg/m^2^ 3 weekly. After 3 cycles the CT scan was repeated to assess the clinical response. Those patients who had partial or complete response were re-assessed by otolaryngologist and were planned for surgery if disease became resectable while other patients were referred for concomitant chemoradiation therapy. 

SPSS version 23 was used to analyze data. Mean and SD was reported for all continuous variables whereas frequencies and percentages were computed for all qualitative variables. Chi square test was applied to see the difference of outcome among effect modifiers. P<0.05 was taken as statistically significant.

**Table 1:** Socio-Demographic Factors

Parameter	n (%)
Age	
< 45 years	45 (45)
=> 45 years	55 (55)
Mean(SD)	41.56 (10.62)
Gender	n (%)
Male	77 (77)
Female	23 (23)
Occupation	n (%)
Employed	74 (74)
Unemployed	26 (26)
Ethnicity	n (%)
Sindhi	26 (26)
Balochi	8 (8)
Punjabi	2 (2)
Pushto	6 (6)
Urdu	58 (58)
Smoking	n (%)
Yes	30 (30)
No	70 (70)

**Table 2:** Clinicopathological Characteristics

Primary tumor site	n (%)	Clinical Stage	n (%)
Lip	3 (3)	Stage 3	4 (4)
Buccal Mucosa	48 (48)	Stage 4	96 (96)
Tongue	19 (19)	Histology	n (%)
Nasopharyngeal	16 (16)	Well Differentiated	25 (25)
Larynx	9 (9)	Moderately differentiated	58 (58)
Hypophyanx	5 (5)	Poorly differentiated	17 (17)
Family history of carcinoma	n (%)		
Yes	8 (8)		
No	92 (92)		

**Table 3:** Stratification of Outcome with Respect to Effect Modifiers

Variables	Outcome					
	Complete Response	Partial Response	No Response	Progression	Total	P-value
Age groups						
<45 years	3	24	2	16	45	0.021
=>45 years	9	38	2	6	55	
Gender						
male	12	43	1	21	77	0.001
female	0	19	3	1	23	
Primary diagnosis						
Lip	0	0	3	1	4	
Buccal Mucosa	6	28	0	13	47	
Tongue	0	13	0	6	19	
Nasopharyngeal	3	12	1	0	16	0.001
Larynx	3	6	0	0	9	
Hypophyanx	0	3	0	2	5	
Histology						
Well Differentiated	3	8	3	11	25	0.001
Moderately differentiated	9	38	0	11	58	
Poorly differentiated	0	16	1	0	17	
Clinical Stage						
III	0	4	0	0	4	0.466
IV	12	58	4	22	96	
Treatment						
CCRT	12	45	3	5	65	
Surgery	0	17	1	2	20	0.001
Best supportive care	0	0	0	15	15	

## Results

Total of 100 patients were included in the study. The age of the patients was 41.56±10.62 years. The majority of the patients were males (77%) whereas 23% were females. Most of them were employed (74%), urdu speaking (58%) and smokers (70%) Table 1.

The buccal mucosa was the most common site in 48 patients. About 58 of the tumors were moderately differentiated and 92 patients didn’t have family history of cancer. According to stage of cancer, 96 patients were identified in stage IV as shown in Table 2. 

Concurrent chemoradiotherapy was performed in majority of the patients (65%) followed by surgery (20%) and best supportive care (15%). The partial response was achieved in majority of the patients after induction chemotherapy with docetaxel and cisplatin regimens (62%). However 22 patients showed progression of disease, 12 patients achieved complete response and only 4 patients showed stable disease (no response). The most frequent side effects due toxicity of chemotherapy with docetaxel and cisplatin regimens were reported as diarrhea (62%) and neutropenia (57%).

The outcome i.e. response rate was stratified with respect to age groups, gender, primary diagnosis, histological type, stage and treatment. All the variables showed statistically significant difference (p<0.05) with respect to outcome except stage of tumor Table 3.

## Discussion

In combination or as a single agent docetaxel is highly active drug for the HNCs. The Phase I trial conducted at Japan showed that endorsed dose of docetaxel was 60 mg/m^2^ and tolerated maximum dose was 70-90 mg/m^2 ^(Kohno, 2005). Docetaxel and cisplatin in combination showed overall response rate as 33-70% for metastatic and recurrent HNCs (Specht et al., 2000; Glisson et al., 2002; Posner and Lefebvre, 2003; Kohno, 2005). In the current study we have evaluated the role of cisplatin plus docetaxel in the treatment of locally advanced HNCs.

In current study, the mean age of HNC patients was 41.56 ± 10.62 years and majority of the patients were of age ≥ 45 years. In a study author found most of the patients with HNCs were in age group of 41-70 years (Larizadeh et al., 2014). In another study author found majority of the patients were in age group =>45 years (56.8%) (Akhtar et al., 2016). Hence, age is a potential factor and with increasing age odds of having HNCs also increased as contrast with young individuals. In the current study majority of the HNCs patients were males (77%). Similar results have been observed in other researches (Bhatia and Jha, 1982; Faggons et al., 2015). In the current study majority of the patients were employed. In a previous study about 239 patients were employed at the time of their diagnosis out of 666 HNCs patients, among them 38.1% of the patients discontinued their job post therapy due to their malignancy and its treatment (Buckwalter et al., 2007). In our study majority of the patients were urdu speaking (58%) followed by sindhi (26%). A Pakistani study reported the HNCs as the most common cancer in Sindhi males and females (8% and 5%) (Khaliq et al., 2013). In the current study about 30% of the patients were smokers. Thus, it has been observed in literature that higher rate of smokers contribute to the incidence of HNCs (Larizadeh et al., 2014). 

In the current study majority of the patients had primary site as buccal mucosa (48%) followed by tongue (19%) and nasopharynx (16%). However, in 2018 a study conducted found the most common sites as nasopharynx (27.9%), larynx (20.5%), sinonasal (12.3%) and oropharynx (12.3%) (Adoga et al., 2018). In the retrospective analysis of HNCs conducted at “Shaukat Khanum Memorial Cancer Hospital and Research Center”, found oral cavity as the most frequent site (43%), among them 9.5% of the cases had buccal mucosa (Faisal et al., 2017). In the current study majority of the HNCs patients had no family history of carcinoma hence another author also showed no association of family history with advanced stage tumor (Adoga et al., 2018). In the present study majority of the patients presented with stage 4 of tumor, similarly in a study conducted by Singh MP et al. found majority of the patients had stage 4 (59.4%) followed by stage 3 in 32.4% of the HNCs patients (Singh et al., 2016). We found moderately differentiated histology in most of the patients (58%) followed by well (25%) and poorly differentiated (17%). Similar findings were revealed by a prveious study, 55% of the patients had moderately differentiated, 24% had well and 21% had poorly differentiated histology of squamous cell carcinoma (Khan et al., 2016). 

In our study patients with locally advanced HNCs were given 3 cycles of induction chemotherapy with docetaxel and cisplatin regimens both at a dose of 75mg/m^2^ 3 weekly. After 3 cycles the CT scan was repeated to assess the clinical response. Majority of the patients achieved partial response after induction of chemotherapy with Docetaxel and Cisplatin (62%), 22% patients showed progression of disease, 12% patients achieved complete response and only 4% patients showed no response. In previous study, author in their trial of docetaxel and cisplatin included 25 HNCs patients who received 5 treatment cycles ranging from 2 to 8 cycles, 8% patients showed complete and 25% showed partial response rate, with overall response rate as 33%. The toxicity was well tolerated and only one patient died due to infection and neutropenia and 3 patients discontinued their treatment due to side effect such as myocardial infraction, massive oedema and persistent thrombocyotopenia (Specht et al., 2000). In the present study, toxicity was also well tolerated and showed adverse events as diarrhea in majority of the patients (62%) followed by neutropenia (57%), vomiting (46%), thromobocytopenia (6%), anemia (4%) and fibrile (2%). A phase II study of weekly docetaxel and cisplatin treatment with concurrent radiotherapy (RT) among 41 patients presented with locally advanced HNCs among them 39.9% achieved complete response after 1 month of induction chemotherapy, moreover 39% of the patients showed mucositis followed by neutropenia (10%) and dysphagia (5%) as adverse events due to toxicity (Lee et al., 2017). Another author evaluated the impact of induction chemotherapy with modified dose of docetaxel, cisplatin, plus 5-fluorouracil followed by concurrent chemotherapy in 52 HNCs patients of Asia and achieved partial response in 60% and complete response in 13.5% of the individuals after induction chemotherapy, moreover 42.3% had complete and 25 had partial response rates following radiotherapy and salvage surgery respectively. The most frequently observed adverse events in their study were neutropenia (35%), stomatitis (35%), anemia (25%), diarrhea (16%), and infections (13.5%) (Wang et al., 2017). 

In our study, the response rate was stratified with respect to age groups, gender, site, histology, stage and treatment. All the variables showed statistical difference (p<0.05) with respect to response rate except stage of tumor. Thirty eight patients had achieved partial response who were age=>45 years and only 9 achieved complete response. About 43 male patients achieved partial response and only 19 achieved complete response. Majority of the patients with buccal mucosa as tumor site achieved partial response and only 6 patients achieved complete response. About 38 patients achieved partial response who had moderately differentiated tumor histology, furthermore 58 patients presented with clinical stage 4 achieved partial response. Majority of the patients who had received concurrent chemoradiotherapy (CCRT) achieved partial response in the present study. In a previous study authors found age, gender, treatment group, vomiting, diarrhea and tumor stage as possible potential factors for HNC patients (Zeng et al., 2018). In another research tolerability and effect of induction chemotherapy with weekly docetaxel, cisplatin, and S-1 (weekly TPS) was evaluated which showed 80% had stage IV disease, while only 20% had stage III disease, among them 25.7% achieved complete response and 60% achieved partial response with overall response rate as 86%, however response rates increased post CCRT as 63% had complete response and 23% had partial response rate (Bae et al., 2013). 

In conclusion, in the present study achievement of partial response was comparatively higher as compared to complete response rate after induction chemotherapy with docetaxel and cisplatin regimens. So it is considered as an effective therapy for the treatment of locally advanced head and neck carcinoma patients and should be suggested for better outcomes.

## Ethical Consideration

Ethical approval was obtained from The Institutional Ethics Committee (No-F.2-81/2018-GENL/Q130/JPMC). Written informed consent from the participants was obtained by the researcher prior to recruitment. Confidentiality of the participants was maintained at all times. Names of the participants were coded by numbers for the privacy. 
